# The relationship between maternal adiposity during pregnancy and fetal kidney development and kidney function in infants: the *Gomeroi gaaynggal* study

**DOI:** 10.14814/phy2.14227

**Published:** 2019-09-13

**Authors:** Yu Qi Lee, Eugenie R. Lumbers, Christopher Oldmeadow, Clare E. Collins, Vanessa Johnson, Lyniece Keogh, Kathryn Sutherland, Adrienne Gordon, Roger Smith, Kym M. Rae, Kirsty G. Pringle

**Affiliations:** ^1^ Priority Research Centre in Reproductive Sciences University of Newcastle Callaghan New South Wales Australia; ^2^ School of Biomedical Sciences and Pharmacy Faculty of Health and Medicine University of Newcastle Callaghan New South Wales Australia; ^3^ Clinical Research Design and Statistical Services Hunter Medical Research Institute University of Newcastle Callaghan New South Wales Australia; ^4^ Priority Research Centre in Physical Activity and Nutrition University of Newcastle Callaghan New South Wales Australia; ^5^ School of Health Sciences Faculty of Health and Medicine University of Newcastle Callaghan New South Wales Australia; ^6^ Gomeroi gaaynggal Centre Faculty of Health and Medicine University of Newcastle Tamworth New South Wales Australia; ^7^ Charles Perkins Centre University of Sydney Sydney Australia; ^8^ School of Medicine and Public Health Faculty of Health and Medicine University of Newcastle Callaghan New South Wales Australia; ^9^ Department of Rural Health University of Newcastle Tamworth New South Wales Australia; ^10^ Priority Research Centre for Generational Health and Ageing University of Newcastle Callaghan New South Wales Australia

**Keywords:** Kidney, maternal obesity, offspring, pregnancy

## Abstract

Maternal obesity during pregnancy has a detrimental impact on offspring renal development and function. This is pertinent to Indigenous Australians as they are twice as likely as non‐Indigenous Australians to develop chronic kidney disease (CKD). The aim of this study was to examine whether there was an association between maternal adiposity and fetal kidney growth in late gestation (>28 weeks) and kidney function in infants, <2.5 years of age, from the *Gomeroi gaaynggal* cohort. Pre‐pregnancy body mass index (BMI) was recorded at the first prenatal visit and maternal adiposity indicators (percent body fat and visceral fat area) measured at >28 weeks gestation by bioelectrical impedance analysis. Fetal kidney structure was assessed by ultrasound. Renal function indicators (urinary albumin:creatinine and protein:creatinine) were measured in infants from a spot urine collection from nappies. Multiple linear regression and multi‐level mixed effects linear regression models with clustering were used to account for repeated measures of urine. 147 mother–child pairs were examined. Estimated fetal weight (EFW), but not fetal kidney size, was positively associated with maternal adiposity and pre‐pregnancy BMI. When adjusted for smoking, combined kidney volume relative to EFW was negatively associated with maternal percentage body fat. Infant kidney function was not influenced by maternal adiposity and pre‐pregnancy BMI (*n* = 84 observations). Current findings show that Indigenous babies born to obese mothers have reduced kidney size relative to EFW. We suggest that these babies are experiencing a degree of glomerular hyperfiltration in utero, and therefore are at risk of developing CKD in later life, especially if their propensity for obesity is maintained. Although no impact on renal function was observed at <2.5 years of age, long‐term follow‐up of offspring is required to evaluate potential later life impacts.

## Introduction

Globally, being overweight or obese is common, based on a recent analysis of 1698 studies that include 19.2 million people across 200 countries (NCD Risk Factor Collaboration 2016). Obesity rates are increasing in rural regions worldwide (NCD Risk Factor Collaboration 2019). The prevalence of being overweight or obese among women of childbearing age is increasing. In the United States of America (USA) and the United Kingdom (UK), more than 50% of women of reproductive age are overweight or obese, and 20–40% of women have exceeded the recommended weight gain during pregnancy (Thangaratinam et al. 2012; Ng et al. 2014). From the time periods 1990–1994 to 2010–2014 in Australia, the prevalence of overweight increased from 12.7% to 16.4% and obesity from 4.8% to 7.3% among 42, 582 nulliparous women (Cheney et al. 2018). Indigenous Australian mothers were 1.6 times more likely to be overweight or obese compared to non‐Indigenous Australian mothers (Australian Institute of Health and Welfare 2016).

It is widely accepted that the prenatal environment plays a crucial role in determining future offspring health (Baird et al. 2017). This concept is commonly referred to as the Developmental Origins of Health and Disease (DOHaD) hypothesis (Barker 2004; Baird et al. 2017). Evidence from both human and animal studies suggests that maternal obesity has a major influence on the risk of chronic disease in offspring, including cardiovascular disease (CVD), hypertension, obesity, type 2 diabetes and metabolic syndrome (Forsen et al. 1997; Laitinen et al. 2001; Barker 2004; Filler et al. 2008; Schack‐Nielsen et al. 2010; Reynolds et al. 2013; Eriksson et al. 2014; 2015; Wankhade et al. 2016). However, in humans, limited studies have specifically evaluated the effect of maternal obesity on offspring renal health and their risk of chronic kidney disease (CKD) (Lee et al. 2019). CKD is one of the fast rising diseases, affecting over 10% of the population globally (Hill et al. 2016). It affects 10% of Australians adults and contributes to one in nine deaths (Australian Institute of Health and Welfare 2018).

Australia's Indigenous people are experiencing an epidemic of renal disease and chronic renal failure. CKD contributes significantly to the disparity in life expectancy between Indigenous and non‐Indigenous Australians (Australian Institute of Health and Welfare 2015). In 2008–2012, 16% of Indigenous deaths were due to CKD (Australian Institute of Health and Welfare 2015), which is 3.5 times higher than the rate for non‐Indigenous Australians. Results from the 2012–2013 National Aboriginal and Torres Strait Islander Health Measures Survey (NATSIHMS) indicate that after adjusting for age differences, Indigenous Australians are twice as likely as non‐Indigenous Australians to develop CKD (Australian Institute of Health and Welfare 2015). In the Northern Territory, Hoy *et al*. reported that Indigenous people have high rates of microalbuminuria, a biomarker for early onset of kidney failure (Hoy et al. 1998; 2005; Hoy and McDonald 2004). Nephron endowment in the Australian Indigenous population was found to be significantly reduced compared to non‐Indigenous people (Douglas‐Denton et al. 2006) and may be linked to the higher incidence of LBW and/or prematurity (14% vs. 9%) in this population (Smith et al. 2000; Rousham and Gracey 2002). Renal biopsies of Indigenous Australian babies of LBW identified lower nephron numbers and larger glomerular volumes (Hughson et al. 2003; Hoy et al. 2006; 2012). Thus, the higher prevalence of LBW and/or preterm Indigenous babies may, through the effects of nephron endowment, be in part responsible for not only the greater prevalence of CKD and end stage kidney disease (ESKD), but also for the prevalence of hypertension and CVD in Indigenous people (Australian Institute of Health and Welfare 2015).

One population‐based, case–control study in 1994 patients with childhood CKD (<21 years of age at diagnosis) and 20,032 controls investigated the association between maternal obesity and risk of CKD in children (Hsu et al. 2014) and demonstrated that higher maternal pre‐pregnancy Body Mass Index (BMI), independent of maternal smoking, high blood pressure and gestational diabetes mellitus (GDM) was associated with a significantly greater risk of renal malformations (dysplasia/aplasia and obstructive uropathy) and childhood CKD compared to controls (24% and 26% increased risk respectively). These data suggest that maternal obesity may be an important prenatal factor for kidney development and predisposition to CKD in adulthood. However, this is the only human study to date that has investigated the potential contribution of maternal obesity to CKD, highlighting that more research is needed (Lee et al. 2019).

The developing kidney is particularly vulnerable to metabolic insults during the critical window of nephrogenesis, with alterations in gene and protein expression proposed as mechanisms underlying the developmental programming of kidney structure and function (Moritz et al. 2008). These alterations lead to a reduction in kidney weight, kidney volume and/or reduction in nephron numbers (Moritz et al. 2008). A lower nephron number and therefore a reduced glomerular filtration surface area may lead to glomerular hyperfiltration and systemic hypertension, causing glomerulosclerosis and progressive renal damage (Brenner et al. 1988; Brenner and Chertow 1994).

In humans, nephrogenesis begins during the ninth week of gestation and is completed at about 36 weeks gestation (Bertram et al. 2011). Therefore, nephron number at birth is greatly influenced by the intrauterine environment and gestational age. Numerous studies of infant kidneys have found that an adverse intrauterine environment (e.g., poor nutrition) results in low birth weight (LBW), preterm birth or intrauterine growth restriction (IUGR). Further, it has been associated with a reduction in nephron endowment (Hinchliffe et al. 1992; 1993; Manalich et al. 2000; Hughson et al. 2003; Rodríguez et al. 2004). However, these studies were conducted on autopsy samples as counting nephron number and/or measuring glomerular size by noninvasive methods is not feasible in humans. Even though kidney weight has been shown to correlate with nephron number (Nyengaard and Bendtsen 1992; Lelievre‐Pegorier and Merlet‐Benichou 2000), kidney weight cannot be measured during intrauterine life. Kidney volume measured by ultrasound and birth weight have been recognized as a valid surrogate measure of nephron number in humans (Hughson et al. 2003; Hughson et al. 2006; Tsuboi et al. 2014). Babies born LBW have fewer nephrons, with a strong correlation between renal volume and nephron number shown in neonates (Hinchliffe et al. 1992; Spencer et al. 2001; Kandasamy et al. 2014; Roderick et al. 2016).

The number of children exposed to an “obesogenic intrauterine environment” during fetal development is increasing in line with the rise in obesity prevalence among women of reproductive age, and this is particularly so in the Indigenous population. Indigenous mothers are 1.6 times more likely to be obese with BMI ≥ 30.0 kg/m^2^ than non‐Indigenous mothers (33% vs. 20%) (Australian Institute of Health and Welfare 2016). Our recent systematic review of human studies found that the influence of maternal obesity on offspring kidney development and kidney function in infants has not been extensively investigated (Lee et al. 2019).

Therefore, the objective of this study was to determine, in an Indigenous Australian population, the associations between maternal adiposity and fetal kidney development in late gestation (>28 weeks) and kidney function in infants, <2.5 years of age.

## Methods

### Setting

This study was embedded within the *Gomeroi gaaynggal* study, a prospective longitudinal cohort of Indigenous Australian mother–child dyads followed from pregnancy, through the postnatal period and up until the children are 10 years of age. The primary site of the study is Tamworth, a rural town in New South Wales, Australia. Further details of the *Gomeroi gaaynggal* study have been published elsewhere (Ashman et al. 2016).

### Ethics

Ethical approval for the study was obtained from the following committees: Hunter New England Human Research Ethics Committee (HNEHREC No. 08/05/21/4.01); the New South Wales Human Research Ethics Committee (NSW HREC HREC/08/HNE/129); and the Aboriginal Health and Medical Research Council Human Research Ethics Committee (AHMRC HREC 654/08).

### Recruitment

Recruitment began in 2010 and is still ongoing at the time of publication. Eligible pregnant Indigenous women are recruited by Indigenous research assistants who attend antenatal clinics at Tamworth Rural Referral Hospital, in New South Wales, Australia. Pregnant women who identify as Indigenous Australians, or as pregnant non‐Indigenous women with Indigenous partners are eligible to participate and can enrol at any stage in their pregnancy. The women provided written informed consent to participate in the study.

### Study design

Pregnancy study visits were carried out in early‐ (<13 weeks), mid‐(13–28 weeks), and late‐pregnancy (>28 weeks). Gestational age was determined by ultrasound at the first study visit. At each visit, various assessments were undertaken including questionnaires, dietary assessments, physical examinations, biological samples (maternal blood and urine), and fetal ultrasounds. Women were asked to complete a self‐reported questionnaire at their first antenatal visit, in which information on Indigenous status, age at recruitment, educational level, cigarette smoking during pregnancy, pre‐pregnancy weight, their obstetric history, and other preexisting medical conditions, including diabetes, asthma, hypertension, and kidney disease were collected.

Follow‐up visits with infants occurred at 3, 6, 9, and 12 months in the first year of life and annually until the child reached 10 years. At each follow‐up visit, various assessments were undertaken including questionnaires, dietary assessments, physical examinations, and biological samples (maternal blood and urine, offspring urine).

### Maternal anthropometry and body composition

Maternal pre‐pregnancy Body Mass Index (BMI) was calculated from measured height (ht) and self‐reported pre‐pregnancy weight (wt) at their first visit during pregnancy [wt(kg)/ht(m^2^)] and each participant was subsequently categorized as being underweight (BMI < 18.5 kg/m^2^), normal weight (BMI 18.5–24.9 kg/m^2^), overweight (BMI 25.0–29.9 kg/m^2^) or obese (BMI ≥ 30.0 kg/m^2^) according to World Health Organization definitions (World Health Organisation 2016). Maternal height was measured without shoes to the nearest 0.1 cm using a wall‐mounted stadiometer and a headboard (model 0123; Seca, Germany) with the head positioned in the Frankfort plane. For participants who could not recall their pre‐pregnancy weight, pre‐pregnancy BMI was calculated using body weight measured prior to 12 weeks’ gestation.

Maternal body composition and weight were measured in a standardized way at each pregnancy study visit using the InBody 720^TM^ multifrequency bioelectrical impedance scales (Biospace Co., Seoul, South Korea), which has 4 pairs of electrodes (octapolar technology) embedded into the handles (thumb and palm electrodes) and floor scale (ball of foot and heel electrodes). It is an inexpensive, rapid, non‐invasive bioelectrical impedance analysis (BIA) method for estimating body composition, that is, weight, BMI, waist:hip ratio (WHR), total body water (TBW), lean body mass, total percentage body fat (PBF), and visceral fat mass (VFM). Participants were asked to remove any jewellery and heavy clothing items and electrodes were cleaned before each participant. Participants step onto the foot electrodes barefoot and grasp the hand electrode cables, with arms held approximately 15º away from the body. Only the maternal body composition measurements taken at the same visit in which an ultrasound was done in the third trimester were used in this analysis.

### Ultrasound measurements

#### Fetal biometry

Fetal biometrics were measured at the same visit as the fetal kidney measurements were made. Ultrasound examinations were performed using a Phillips Cx50 Portable Diagnostic Ultrasound with a 5 MHz convex transducer and used to determine gestational age and fetal growth. During each ultrasound examination, fetal biometry including head circumference, abdominal circumference, and femur length were measured. Estimated fetal weight (EFW) was calculated using the formula by Hadlock using head circumference (HC), abdominal circumference (AC) and femur length (FL): EFW (g) = 10 × (1.326 − 0.00326 × AC × FL + 0.0107 × HC + 0.0438 × AC + 0.158 × FL) (Hadlock et al. 1985).

#### Kidney measurements

Fetal kidney size and volume were measured by ultrasound by determining the length, the anterior–posterior diameter (thickness) and the transverse diameter (width). Renal length was the maximum longitudinal length measured. Anterior–posterior diameter was measured as the maximum distance between the anterior and posterior wall of the kidney. The transverse diameter was the maximum transverse diameter on a transverse scan. Anterior–posterior and transverse kidney diameter were measured perpendicular to each other, outer to outer, above the hilum (Dinkel et al. 1985). Kidney volume was calculated using the formula for an ellipsoid: volume (cm^3^) = length (mm) × transverse (mm) × anterior–posterior (mm) × 0.523) (Jeanty et al. 1982). Combined kidney volume (cm^3^) was calculated as the sum of the left and right kidney volume. Relative combined kidney volume was calculated as the ratio of combined kidney volume/EFW (cm^3^/kg). Relative kidney measurements better represent kidney size than absolute kidney measurements as it eliminates sex and length differences (Konje et al. 1997; Gloor et al. 1997). For this study, only the kidney measurements taken in the third trimester were used in the analysis.

### Pregnancy and delivery outcomes

Maternal pregnancy and birth outcomes including date of birth, birth weight, gestational age, newborn body measurements, and child gender were obtained from patient records.

### Offspring kidney function

Kidney function was determined by urinary albumin:creatinine and protein:creatinine measured in infants after spot urine collection from nappies. For the purpose of this analysis, we have only included data from follow‐up visits with infants up until the age of 2.5 years.

### Population for analysis

Twin pregnancies (*n* = 7) were excluded from the analysis. Those with implausible maternal body composition measurements attributed to equipment issues when measuring were also excluded (*n* = 4).

Only those with third trimester kidney ultrasound and third trimester maternal body composition measurements available were included in the initial analysis. This analysis was performed in a total of 147 mother–child dyads. In the follow‐up analysis, only those with a kidney function measurement in infancy and a third trimester maternal body composition measurements were included (*n* = 57). There were multiple follow‐up time‐points (at 3, 6, 9, 12 months and 2 years) and individuals with repeated urinary measures were included (*n* = 84 observations).

### Statistical analysis

Continuous data were summarized using descriptive statistics including the number of observations used in the calculation (*n*) and were tested for normality, with normally distributed data reported as mean (95% CI) and non‐normal data reported as median [IQR]. Categorical data were summarized as counts and percentages of each category.

Multiple linear regression models were used to assess how much the maternal adiposity during pregnancy (percentage body fat and visceral fat) and pre‐pregnancy BMI explain the variation in fetal kidney structural development (length, anterior–posterior, transverse and volume) in the third trimester of pregnancy. Multi‐level mixed effects linear regression models with clustering were used to account for repeated urinary measures in infants. To assess the robustness of the current results, several sensitivity analyses were carried out. Content knowledge was utilized to select covariates.

Covariates tested were fetal sex, gestational age in days, smoking, diabetes mellitus (type 1, type 2 or GDM), and hypertensive disorders (chronic hypertension, gestational hypertension or preeclampsia). We adjusted for covariates that changed the effect estimate of maternal percentage body fat, maternal visceral fat, and pre‐pregnancy BMI on fetal kidney structure by more than 15% when added to the baseline model. The model focusing on the fetal kidney structural parameters was adjusted for fetal sex, gestational age in days, and smoking. The model focusing on the relative fetal kidney structural parameters was adjusted for smoking only. Pregnancy complications (diabetes mellitus and hypertensive disorders during pregnancy) did not change the effect estimate by more than 15%, thus were not including in the final model.

Further analyses were done by grouping the mothers according to their pre‐pregnancy BMI: non‐obese group (underweight + normal pre‐pregnancy BMI) and obese group (overweight + obese pre‐pregnancy BMI). The relative combined fetal kidney volume between the two groups were then compared and represented graphically as side‐by‐side box plots. *T*‐tests were used to evaluate relative combined fetal kidney volume between nonobese and obese groups.

All data manipulation and statistical analyses were performed using the statistical software package Intercooled Stata, version 14 (Stata Corp LP, College Station, TX). All measures of association are presented with their 95% confidence intervals (95% CI). A 2‐sided *P* value of <0.05 was considered statistically significant. Adjusted R^2^ values and coefficients (95% CI) are reported, with *R* ^2^ ⩾0.26 considered large, ⩾0.13 to  <0.26 medium and ⩽0.02 small.

## Results

Cohort characteristics of this population of Indigenous Australian pregnant women with singleton pregnancies are summarized in Table 1. The median maternal age was 23.6 years (IQR: 20.5, 29.0) at time of consent to participate (*n* = 147). Of those who self‐reported their prepregnancy weight, 5.5% (*n* = 6/108) of mothers were underweight (BMI < 18.5 kg/m^2^), 31.5% (*n* = 34/108) were within the normal weight range (BMI 18.5–24.99 kg/m^2^) and 63% (*n* = 68/108) were overweight/obese (BMI⩾25.0 kg/m^2^). The proportion who self‐reported smoking during pregnancy was 38.4% (*n* = 56/146). 2.7% of women in the cohort (*n* = 3/112) had preexisting type 1 diabetes, none had diagnosed preexisting type 2 diabetes, 13.6% (*n* = 15/110) developed GDM, 0.9% (*n* = 1/112) had chronic hypertension, 6.3% (*n* = 7/111) had gestational hypertension and 10% (*n* = 11/110) developed preeclampsia during their pregnancy. The majority of the women had a high school education or less (72%).

**Table 1:** Maternal characteristics.

Variables	*n* (%)
Indigenous status (*n* = 147)	
Indigenous	120 (81.6)
Carrying an Indigenous child	27 (18.4)
Educational level (school attainment) (*n* = 100)	
<Year 10	13 (13)
Year 10 or equivalent	38 (38)
Year 12 or equivalent	21 (21)
Trade/apprenticeship	17 (17)
Undergraduate degree	4 (4)
Post‐graduate degree	2 (2)
Currently studying	5 (5)
Pre‐pregnancy BMI status (kg/m^2^) (*n* = 108)	
Underweight (<18.5 kg/m^2^)	6 (5.5)
Normal weight (18.5–24.99 kg/m^2^)	34 (31.5)
Overweight/obese (≥25.0 kg/m^2^)	68 (63.0)
Number with diabetes mellitus	
Type 1 (*n* = 112)	3 (2.68)
Type 2 (*n* = 112)	0
Gestational diabetes (*n* = 110)	15 (13.6)
Number with hypertensive disorders	
Chronic hypertension (*n* = 112)	1 (0.9)
Gestational hypertension (*n* = 111)	7 (6.3)
Preeclampsia (*n* = 110)	11 (10)
Smoked during pregnancy (*n* = 146)	
Yes (at any point during pregnancy)	56 (38.4)

BMI, body mass index.

We assessed maternal adiposity during the third trimester and used measured percentage body fat and visceral fat area as adiposity indicators. The median percentage body fat of Indigenous pregnant women in this cohort was 43% (IQR: 35.4, 50, *n* = 147) and the median visceral fat area was 142.8 cm^2^ (IQR: 102.7, 205.5, *n* = 147). Both maternal adiposity and fetal kidney ultrasound were measured at a median gestational age of 35.3 weeks.

Of those with fetal sex information available (*n* = 126), 52 babies were female (41.2%) and 74 were male (58.7%). Of those with birth outcome data available (*n* = 113), 102 babies were born at term (>37 weeks) and 11 were born prematurely (9.7%), with a median age at birth of 35.9 weeks (IQR: 35.6, 36.4). Of those included in the follow‐up analysis of kidney function in infants at < 2.5 years old (*n* = 57), 22 infants were female (38.6%) and 35 were male (61.4%).

Table 2 (adjusted for smoking, gestational age in days, fetal sex) and Table S1 (https://figshare.com/s/b63c3d57549d5164a0af) (unadjusted) give the associations of maternal percentage body fat, visceral fat area in the third trimester and prepregnancy BMI with EFW and fetal kidney structural parameters (length, anterior–posterior diameter, transverse diameter and volume) measured in late pregnancy.

**Table 2:** Third trimester maternal adiposity and its associations with fetal kidney structural outcomes in the third trimester.

	Maternal Percent Body Fat					Maternal Visceral Fat Area (cm^2^)					Pre‐pregnancy BMI (kg/m^2^)				
	*n*	Coefficient	95% CI	*R* ^2^	*P*	*n*	Coefficient	95% CI	*R* ^2^	*P*	*n*	Coefficient	95% CI	*R* ^2^	*P*
EFW (kg)	126	0.01	0.003, 0.02	0.79	0.003	126	0.002	0.001, 0.002	0.82	<0.001	94	0.01	0.003, 0.02	0.78	0.005
Fetal Left kidney structures															
Length (mm)	126	−0.02	−0.1, 0.07	0.23	0.71	126	−0.001	−0.01, 0.01	0.23	0.81	94	−0.02	−0.12, 0.08	0.23	0.74
Anterior‐posterior (mm)	126	−0.02	−0.08, 0.04	0.14	0.42	126	0.002	−0.005, 0.008	0.14	0.65	94	0.01	−0.06, 0.09	0.11	0.70
Transverse (mm)	126	0.02	−0.04, 0.08	0.25	0.57	126	0.0005	−0.006, 0.007	0.24	0.89	94	0.07	−0.009, 0.14	0.28	0.08
Left kidney volume (cm^3^)	126	−0.01	−0.07, 0.05	0.28	0.71	126	0.0002	−0.006, 0.006	0.28	0.96	94	0.02	−0.05, 0.10	0.26	0.50
Fetal Right kidney structures															
Length (mm)	125	0.01	−0.08, 0.11	0.14	0.77	125	0.007	−0.003, 0.02	0.16	0.17	94	0.05	−0.06, 0.17	0.18	0.35
Anterior‐posterior (mm)	125	0.03	−0.03, 0.10	0.17	0.32	125	0.007	0.001, 0.01	0.20	0.03	94	0.04	−0.03, 0.11	0.23	0.22
Transverse (mm)	125	0.005	−0.06, 0.07	0.24	0.90	125	0.005	−0.002, 0.01	0.25	0.19	94	0.06	−0.02, 0.14	0.30	0.15
Right kidney volume (cm^3^)	125	0.009	−0.05, 0.07	0.27	0.78	125	0.005	−0.001, 0.01	0.28	0.15	94	0.04	−0.03, 0.11	0.33	0.29
Combined kidney volume (cm^3^)	124	−0.003	−0.11, 0.11	0.30	1	124	0.005	−0.007, 0.02	0.30	0.40	93	0.06	−0.07, 0.20	0.32	0.36

EFW, estimated fetal weight; BMI, body mass index; CI, confidence intervals. Adjusted for smoking, gestational age (days), and fetal sex.

In the adjusted model (Table 2), EFW (kg) was positively associated with maternal percentage body fat, visceral fat area in late pregnancy and pre‐pregnancy BMI. Only fetal right kidney anterior–posterior diameter was positively associated with the maternal visceral fat area in the third trimester (*P* = 0.03). The fetal right kidney anterior–posterior diameter increased by 0.007 mm for each Standard Deviation Score (SDS) increase in maternal visceral fat area. Any other measures of fetal kidney size were not associated with maternal adiposity and prepregnancy BMI.

In the unadjusted model (Table S1: https://figshare.com/s/b63c3d57549d5164a0af), fetal kidney structural parameters measured in late pregnancy were not associated with maternal percentage body fat, visceral fat area and prepregnancy BMI.

When adjusted for smoking (Table 3), left fetal kidney volume/EFW and combined fetal kidney volume/EFW were negatively associated with maternal percentage body fat (*P* = 0.04 and *P* = 0.03, respectively). Left fetal kidney volume/EFW and combined fetal kidney volume/EFW decreased by 0.02 and 0.04 cm^3^/kg, respectively, for each Standard Deviation Score (SDS) increase in maternal percent body fat. Additionally, left kidney volume/EFW was negatively associated with maternal visceral fat area only (*P* = 0.03). Fetal kidney volume/EFW (left and right) and combined fetal kidney volume/EFW were not associated with pre‐pregnancy BMI.

**Table 3:** Association between maternal adiposity and kidney volume/estimated fetal weight (EFW) in the third trimester.

	Maternal Percent Body Fat					Maternal Visceral Fat Area (cm^2^)					Pre‐pregnancy BMI (kg/m^2^)				
	*n*	Coefficient	95% CI	*R* ^2^	*P*	*n*	Coefficient	95% CI	*R* ^2^	*P*	*n*	Coefficient	95% CI	*R* ^2^	*P*
Left kidney volume/EFW (cm^3^/kg)	144	−0.02	−0.04, −0.001	0.02	**0.04**	144	−0.002	−0.004, −0.0002	0.02	**0.03**	106	−0.007	−0.03, 0.01	−0.01	0.50
Right kidney volume/EFW (cm^3^/kg)	143	−0.02	−0.04, 0.002	0.03	0.08	143	−0.001	−0.003, 0.001	0.02	0.32	106	−0.01	−0.03, 0.01	0.04	0.35
Combined kidney volume/EFW (cm^3^/kg)	142	−0.04	−0.07, −0.004	0.03	**0.03**	142	−0.003	−0.007, 0.0004	0.01	0.08	105	−0.02	−0.05, 0.02	−0.004	0.38

BMI, body mass index; CI, confidence intervals. Adjusted for smoking. *P* values in bold are statistically significant at *P*<0.05.

In the unadjusted model (Table S2: https://figshare.com/s/9e4cfc826362074ffb6c), fetal kidney volume/EFW (left and right) and combined fetal kidney volume/EFW were negatively associated with maternal percentage body fat (*P* = 0.04, *P* = 0.04 and *P* = 0.02 respectively). Left fetal kidney volume/EFW remained negatively associated with maternal visceral fat area (*P* = 0.03). Fetal kidney volume/EFW (left and right) and combined fetal kidney volume/EFW were not associated with prepregnancy BMI.

**Figure 1:**
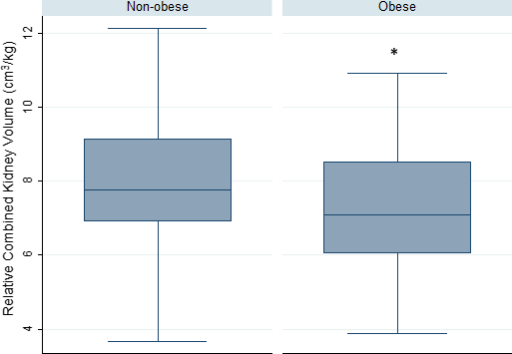
Comparison of third trimester combined fetal kidney volume/estimated fetal weight between infants of non‐obese (underweight + normal pre‐pregnancy BMI) and obese (overweight + obese pre‐pregnancy BMI) mothers. The combined kidney volume to estimated fetal weight ratio of infants born to obese mothers (*n* = 65) was smaller than that of infants born to non‐obese mothers (*n* = 40, *P* = 0.035). Data are presented as medians and interquartile ranges. * denotes *P* < 0.05. BMI, body mass index.

In late pregnancy, fetuses from mothers in the obese group (overweight + obese prepregnancy BMI) had smaller combined fetal kidney volume/EFW ratio (cm^3^/kg) than fetuses from mothers in the nonobese group (underweight + normal prepregnancy BMI) (*P* = 0.035, Fig. 1).

**Figure 2:**
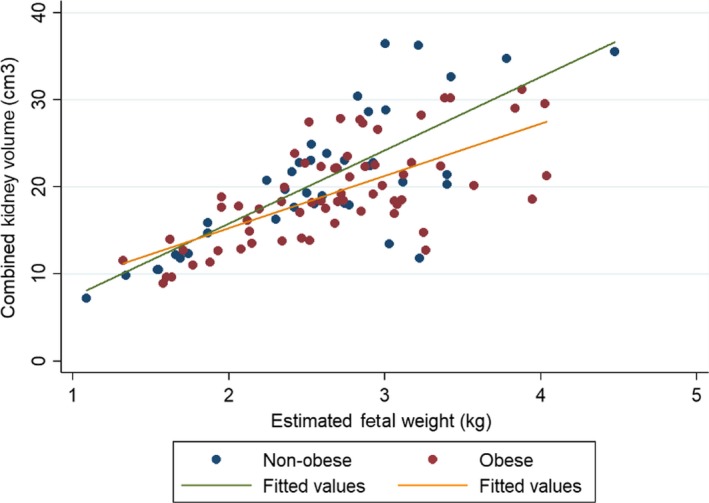
Combined kidney volume of fetuses from non‐obese (underweight + normal pre‐pregnancy BMI) and obese (overweight + obese pre‐pregnancy BMI) mothers. Simple linear regression (non‐adjusted) was used in this model.

There were near significant differences in the association between combined kidney volume and EFW in late pregnancy when comparing mothers in the non‐obese and obese groups (*P* = 0.065). For the non‐obese group, spearman's rho = 0.67, and for the obese group, spearman's rho = 0.66. Figure 2 illustrates the linear regression model of the relationship between fetal combined kidney volume (cm^3^) and EFW (kg) in the non‐obese and obese groups.

Infant kidney function, assessed by measuring urinary albumin:creatinine and protein:creatinine, was not associated with any alterations in maternal percent body fat and visceral fat area in the third trimester and pre‐pregnancy BMI (Table 4, *n* = 84 observations from 57 infants).

**Table 4:** Associations between maternal adiposity in the third trimester, pre‐pregnancy BMI and infant kidney function.

	Maternal Percent Body Fat				Maternal Visceral Fat Area (cm^2^)				Pre‐pregnancy BMI (kg/m^2^)			
	*n*	Coefficient	95% CI	*P*	*n*	Coefficient	95% CI	*P*	*n*	Coefficient	95% CI	*P*
Protein: Creatinine (mg/mmol)	84	0.006	−0.001, 0.01	0.1	84	0.0005	−0.0003, 0.001	0.19	64	0.004	−0.002, 0.01	0.21
Albumin: Creatinine (mg/mmol)	84	0.08	−0.06, 0.21	0.26	84	0.005	−0.008, 0.02	0.48	64	0.03	−0.11, 0.16	0.7

BMI, body mass index; CI, confidence intervals. Adjusted for smoking.

## Discussion

To our knowledge, this is the first study that examines the possible association between maternal adiposity in pregnancy and human fetal kidney growth, and with kidney function in infancy. It is also unique because it has been carried out with an Indigenous Australian population residing in a rural region of New South Wales, Australia. The analysis was performed using fetal kidney ultrasound measurements after 26 weeks of gestation as the period of maximum kidney growth occurs between 26 and 34 weeks of gestation. The current study shows that EFW, but not fetal kidney size in late fetal life, is positively associated with maternal adiposity and pre‐pregnancy BMI. Consequently, fetal kidney volume relative to EFW is negatively associated with maternal adiposity. Furthermore, kidney function in infants <2.5 years old is not associated with maternal adiposity during pregnancy and pre‐pregnancy BMI.

Much of our understanding of the association between maternal obesity and offspring kidney development and subsequent risk of CKD has come from animal models. In a high fat diet‐induced rodent model of obesity, mice were exposed to high‐fat diet six weeks prior to mating, during gestation and lactation, and male offspring of these obese rodent mothers have increased albuminuria, increased serum creatinine levels, increased fibrotic, inflammatory, and oxidative stress changes in the kidneys, reflecting adverse renal pathology (Glastras et al. 2016; 2017). Jackson *et al.* reported glomerulosclerosis and reduced kidney function, including increased urinary albumin excretion, in male rat offspring exposed to a maternal diet high in fructose and fat compared to controls (Jackson et al. 2012). Furthermore, female mice exposed to a maternal high fat/fructose diet had lower glomerular filtration rates (Flynn et al. 2013). Taken together, these studies suggest that a maternal obesogenic diet can have a detrimental impact on the programming of offspring renal development and later renal function.

Only one epidemiological study to date has investigated the impact of maternal obesity on offspring kidney outcomes in humans (Hsu et al. 2014; Lee et al. 2019). As such, there is a gap in our knowledge on the potential contribution of maternal obesity to the current CKD epidemic (Hill et al. 2016), especially in the Indigenous population where the risk of ESKD is higher. The kidney remains a largely unexplored area within the DOHaD field in relation to exposure to an obesogenic intrauterine environment.

The proportion of women of reproductive age who are overweight or obese is rapidly increasing globally and this is becoming an issue, not only in developed countries, but in developing nations as well. Maternal pre‐pregnancy BMI and gestational weight gain (GWG) are strongly associated with an increased risk of most pregnancy complications, such as GDM and preeclampsia (Norman and Reynolds 2011; Santos et al. 2019). Epidemiological studies have shown that exposure to an obesogenic intrauterine environment has potential lasting ramifications for the long‐term health and disease risk in offspring (Catalano and Ehrenberg 2006; Glastras et al. 2018). Yet, limited human studies have investigated whether or not maternal obesity can affect the developing kidney and subsequent renal health (Catalano and Ehrenberg 2006; Richter et al. 2016), which warrants further investigation.

In this study, we did not detect any significant associations between maternal adiposity or pre‐pregnancy BMI with fetal kidney size in late pregnancy when smoking, gestational age and fetal sex were taken into account. This is in line with previous findings from the Generation R study (Verburg et al. 2007) that found that maternal obesity was not associated with combined fetal kidney volume in late pregnancy, even though maternal obesity during pregnancy is known to influence overall fetal body size in humans (Catalano and Ehrenberg 2006).

Exposure to an obesogenic intrauterine environment could lead to an increase in fetal size and fat mass, as well as altered metabolic programming in offspring (Oestreich and Moley 2017). In the current cohort, we found that EFW is positively associated with maternal percentage body fat, visceral fat area in the third trimester and pre‐pregnancy BMI when taking into account smoking, gestational age and fetal sex. Other evidence shows that offspring born to obese mothers have higher birth weights, increased BMI and fat deposition, and an increase in adolescent and adult obesity (Surkan et al. 2004; Catalano and Ehrenberg 2006; Catalano et al. 2009; Pringle et al. 2019).

Additionally, we have shown that a smaller fetal kidney volume relative to EFW is associated with a higher maternal adiposity in late pregnancy and being in the overweight/obese pre‐pregnancy BMI category. In humans, an accurate nephron count can only be done post mortem, however it has been demonstrated that neonatal renal volume is a valid surrogate measure for nephron number (Spencer et al. 2001; Luyckx and Brenner 2010; Kandasamy et al. 2013; 2014). The current findings therefore suggest that in these bigger‐sized fetuses, with smaller kidney volume relative to EFW, there are possibly fewer nephrons relative to body weight. This mismatch between an increased body size and a reduced kidney volume (and presumably nephron mass) relative to body size may result in an increase in the single nephron glomerular filtration rate (D'Agati et al. 2016). Thus, we suggest that Indigenous babies born to obese mothers may already be experiencing compensatory glomerular hyperfiltration *in utero* *,* which leads to glomerular hypertrophy of existing nephrons (Bagby 2009).

In adults, obesity‐induced hyperfiltration is an etiological factor in obesity‐related glomerulonephropathy (Chagnac et al. 2003; Wuerzner et al. 2010; Tsuboi et al. 2017). In the long term, glomerular hypertrophy and sclerosis, which lead to hypertension and progressive kidney damage, may result in further reduction of nephron mass and a vicious cycle of glomerular injury (Magee et al. 2009; Mascali et al. 2016). This would predispose these Indigenous children to a higher risk of CKD in later life. Since babies whose mothers were obese are born bigger and maintain this difference throughout childhood, the current findings suggest that obesity driven glomerular hyperfiltration begins *in utero* and continues throughout life. Thus, exposure to an obesogenic intrauterine environment is an additional risk factor in the predisposition to CKD in adult life.

In a study of kidney autopsies from an adult population without any form of renal disease, it was found that a high BMI and a low total glomerular number were associated with increased glomerular volume (Hoy et al. 2010). In an analysis of renal biopsies from a cohort of patients with CKD, it was demonstrated that a high BMI and a low glomerular density are independently associated with an increased mean glomerular volume (Tsuboi et al. 2013). In addition, the analyses using the glomerular density/BMI ratio demonstrated a close association with the mean glomerular volume (Tsuboi et al. 2013). These results support our mismatch hypothesis, in which an increased body size and a relative reduction in nephron mass result in glomerular enlargement and subsequent renal failure.

In this cohort of Indigenous infants (*n* = 57), it was found that maternal adiposity in the third trimester and pre‐pregnancy BMI had no influence on kidney function in infants < 2.5 years old. In a high‐fat diet‐induced rodent model of obesity, male offspring exhibited elevated albuminuria and serum creatinine levels in adulthood (Glastras et al. 2016; 2017). Therefore, as reflected in animal studies, the detrimental consequences to offspring kidney function in humans when exposed to an obesogenic intrauterine environment may not surface until later in life. It is therefore imperative to investigate whether there are signs of renal damage or detrimental impact on renal function later in life in these babies.

BMI is a surrogate indicator of obesity, does not measure fat distribution and is a poor indicator of variations in maternal fat mass in late pregnancy that potentially influence fetal growth (Sewell et al. 2007). BIA can accurately measure percent body fat composition and fat distribution and is considered a better predictor of maternal nutritional status than BMI (Sanin Aguirre et al. 2004; Widen and Gallagher 2014). In this study, in addition to pre‐pregnancy BMI, we used BIA to further investigate the relationship between maternal fat distribution (percentage body fat and visceral fat area) and fetal kidney development. Although assessment of adipose tissue is less clinically accessible, it is of primary interest, as maternal visceral adipose tissue influences maternal inflammatory responses and the development of metabolic syndrome associated with adiposity (Diamant et al. 2005; Han and Lean 2016). Additionally, the amount of maternal visceral adipose tissue has an adverse effect on metabolic risk, inflammatory responses and insulin resistance in offspring (Long et al. 2012; Alfaradhi et al. 2016).

While the data presented may contribute to a greater understanding of the relationship between maternal adiposity and fetal kidney growth, questions remain about the validity of BIA measurements in pregnancy. However, BIA has been previously shown to accurately predict TBW during pregnancy and validated against other techniques of body composition analysis such as deuterium water and hydrodensitometry (Raaij et al. 1988; Van et al. 1995; McCarthy et al. 2004). There is an increasing number of body composition studies utilizing BIA to estimate fat‐free mass and fat mass of pregnant women (Farah et al. 2011; Kugananthan et al. 2017; Piuri et al. 2017), supporting the applicability of this method in research studies. The use of BIA in pregnancy is a safe and simple technique and may therefore be appropriate for large epidemiological studies.

The strengths of this study include: (1) the detailed analysis of renal ultrasound examinations, (2) accurate measurement of maternal height during the first antenatal visit to enhance accuracy of BIA estimates of body composition, (3) minimization of confounding variables by excluding multiple pregnancies and adjusting for them in our statistical model, (4) direct measurement of maternal body composition, using BIA rather than depending on surrogate measurements such as BMI, and (5) collection of data and clinical information from patient hospital records. One limitation is the relatively small sample size, however, recruitment is still ongoing. There are several reasons for lack of full retention of all women postpartum. While every effort is made to retain all participants, challenges to retention include cohort participants moving away from the study location, time constraints on mothers and the lack of incentives for continued participation.

## Conclusion

This study is the first study to investigate the association between maternal adiposity and human fetal kidney growth, and kidney function in infancy in an Indigenous Australian population. The current findings suggest that in late pregnancy, fetal kidney volume relative to EFW is negatively associated with maternal adiposity. Thus, Indigenous babies born to obese mothers have smaller kidney volumes relative to EFW. Since kidney volume is a surrogate for nephron number, these infants are likely to have fewer nephrons relative to body weight, and these nephrons are likely to be hyperfiltering. However, we are unable to directly measure hyperfiltration and nephron number in these babies in the late pregnancy period. Future studies that focus on maternal body composition, particularly maternal fat mass, and its influence on offspring kidney development and function in a larger Indigenous cohort are warranted to guide the development of future interventions and public health policies to optimize maternal and fetal health. In addition, future studies that include long‐term follow‐up of the children in the current cohort to assess renal damage and blood pressure in offspring are imperative to determine whether and to what extent, exposure to an intrauterine obesogenic environment influences renal function in postnatal life.

## Conflict of Interest

None.

## Supporting information




**Table S1.** Third trimester maternal adiposity and its associations with fetal kidney structural outcomes in the third trimester.

**Table S2.** Association between maternal adiposity and kidney volume/estimated fetal weight (EFW) in the third trimester.
