# Supplementary material for: The relationship between maternal adiposity during pregnancy and fetal kidney development and kidney function in infants: the Gomeroi gaaynggal study
Source: Physiol Rep. 2019 Sep 13;7(17):e14227. doi: 10.14814/phy2.14227 (PMC6742895; doi:10.14814/phy2.14227)
Supplement: Supplementary file 1 — Table S1. Third trimester maternal adiposity and its associations with fetal kidney structural outcomes in the third trimester. [file PHY2-7-e14227-s001.docx]

**Supplementary Table 1: Third trimester maternal adiposity and its associations with fetal kidney structural outcomes in the third trimester.**

**EFW:** estimated fetal weight; **BMI:** body mass index; **CI:** confidence intervals. Unadjusted.

|  | **Maternal Percent body fat** | | | | | **Maternal Visceral Fat Area (cm^2^)** | | | | | **Pre-pregnancy BMI (kg/m^2^)** | | | | |
| --- | --- | --- | --- | --- | --- | --- | --- | --- | --- | --- | --- | --- | --- | --- | --- |
|  | **n** | **Coefficient** | **95% CI** | **R^2^** | **P** | **n** | **Coefficient** | **95% CI** | **R^2^** | **P** | **n** | **Coefficient** | **95% CI** | **R^2^** | **P** |
| EFW (kg) | 146 | 0.01 | -0.003, 0.02 | 0.02 | 0.13 | 146 | 0.001 | -0.00001, 0.003 | 0.03 | **0.05** | 107 | 0.008 | -0.006, 0.02 | 0.01 | 0.27 |
| **Fetal Left kidney structures** | | | | | | | | | | | | | | | |
| Length (mm) | 146 | -0.02 | -0.11, 0.08 | 0.001 | 0.66 | 146 | -0.004 | -0.01, 0.006 | 0.005 | 0.38 | 107 | -0.04 | -0.15, 0.07 | 0.005 | 0.48 |
| Anterior-posterior (mm) | 146 | -0.03 | -0.09, 0.03 | 0.008 | 0.28 | 146 | 0.0001 | -0.006, 0.007 | 0.00 | 0.96 | 107 | 0.006 | -0.07, 0.08 | 0.0002 | 0.88 |
| Transverse (mm) | 146 | 0.02 | -0.04, 0.09 | 0.004 | 0.47 | 146 | -0.0002 | -0.007, 0.007 | 0.00 | 0.96 | 107 | 0.06 | -0.01, 0.14 | 0.03 | 0.1 |
| Left kidney volume (cm^3^) | 146 | -0.01 | -0.07, 0.05 | 0.001 | 0.69 | 146 | -0.001 | -0.008, 0.005 | 0.001 | 0.68 | 107 | 0.01 | -0.06, 0.09 | 0.001 | 0.71 |
| **Fetal Right kidney structures** | | | | | | | | | | | | | | | |
| Length (mm) | 145 | 0.0008 | -0.09, 0.09 | 0.00 | 1 | 145 | 0.003 | -0.006, 0.01 | 0.003 | 0.53 | 107 | 0.02 | -0.09, 0.14 | 0.002 | 0.69 |
| Anterior-posterior (mm) | 145 | 0.008 | -0.05, 0.07 | 0.0004 | 0.80 | 145 | 0.004 | -0.003, 0.01 | 0.01 | 0.25 | 107 | 0.005 | -0.06, 0.07 | 0.0002 | 0.89 |
| Transverse (mm) | 145 | 0.004 | -0.07, 0.08 | 0.0001 | 0.90 | 145 | 0.002 | -0.005, 0.01 | 0.003 | 0.54 | 107 | 0.04 | -0.04, 0.12 | 0.008 | 0.36 |
| Right kidney volume (cm^3^) | 145 | -0.002 | -0.07, 0.06 | 0.00 | 0.94 | 145 | 0.002 | -0.005, 0.01 | 0.002 | 0.64 | 107 | 0.01 | -0.07, 0.09 | 0.0005 | 0.82 |
|  | | | | | | | | | | | | | | | |
| Combined kidney volume (cm^3^) | 144 | -0.01 | -0.13, 0.11 | 0.0002 | 0.87 | 144 | 0.0005 | -0.01, 0.01 | 0.00 | 0.94 | 106 | 0.02 | -0.12, 0.17 | 0.001 | 0.75 |
