# Supplementary material for: The relationship between maternal adiposity during pregnancy and fetal kidney development and kidney function in infants: the Gomeroi gaaynggal study
Source: Physiol Rep. 2019 Sep 13;7(17):e14227. doi: 10.14814/phy2.14227 (PMC6742895; doi:10.14814/phy2.14227)
Supplement: Supplementary file 2 — Table S2. Association between maternal adiposity and kidney volume/estimated fetal weight (EFW) in the third trimester. [file PHY2-7-e14227-s002.docx]

**Supplementary Table 2: Association between maternal adiposity and kidney volume/estimated fetal weight (EFW) in the third trimester.**

|  | **Maternal Percent body fat** | | | | | **Maternal Visceral Fat Area (cm^2^)** | | | | | **Pre-pregnancy BMI (kg/m^2^)** | | | | |
| --- | --- | --- | --- | --- | --- | --- | --- | --- | --- | --- | --- | --- | --- | --- | --- |
|  | **n** | **Coefficient** | **95% CI** | **R^2^** | **P** | **n** | **Coefficient** | **95% CI** | **R^2^** | **P** | **n** | **Coefficient** | **95% CI** | **R^2^** | **P** |
| Left kidney volume/EFW (cm^3^/kg) | 145 | -0.02 | -0.04, -0.001 | 0.03 | **0.04** | 145 | -0.002 | -0.004, -0.0003 | 0.03 | **0.03** | 106 | -0.006 | -0.03, 0.01 | 0.004 | 0.54 |
| Right kidney volume/EFW (cm^3^/kg) | 144 | -0.02 | -0.04, -0.001 | 0.03 | **0.04** | 144 | -0.001 | -0.003, 0.0007 | 0.01 | 0.19 | 106 | -0.01 | -0.03, 0.007 | 0.01 | 0.21 |
| Combined kidney volume/EFW (cm^3^/kg) | 143 | -0.04 | -0.07, -0.006 | 0.04 | **0.02** | 143 | -0.003 | -0.007, -0.00008 | 0.03 | 0.06 | 105 | -0.02 | -0.05, 0.02 | 0.01 | 0.32 |

**BMI:** body mass index; **CI:** confidence intervals. Unadjusted.
